# Supplementary material for: Multi-omics analysis of human mesenchymal stem cells shows cell aging that alters immunomodulatory activity through the downregulation of PD-L1
Source: Nat Commun. 2023 Jul 20;14:4373. doi: 10.1038/s41467-023-39958-5 (PMC10359415; doi:10.1038/s41467-023-39958-5)
Supplement: Supplementary file 1 — Supplementary Information [file 41467_2023_39958_MOESM1_ESM.pdf]

# Supplementary Information

## Multi-omics analysis of human mesenchymal stem cell shows cell aging that alters immunomodulatory activity through the downregulation of PD-L1

Yuchen Gao[#], Ying Chi[#], Yunfei Chen[#], Wentian Wang[#], Huiyuan Li, Wenting Zheng, Ping Zhu, Jinying An, Yanan Duan, Ting Sun, Xiaofan Liu, Feng Xue, Wei Liu, Rongfeng Fu, Zhibo Han, Yingchi Zhang, Renchi Yang, Tao Cheng, Jun Wei[*], Lei Zhang[*], Xiaomin Zhang[*]

**Contents:**

Supplementary Figures 1-8

Supplementary Fig. 1

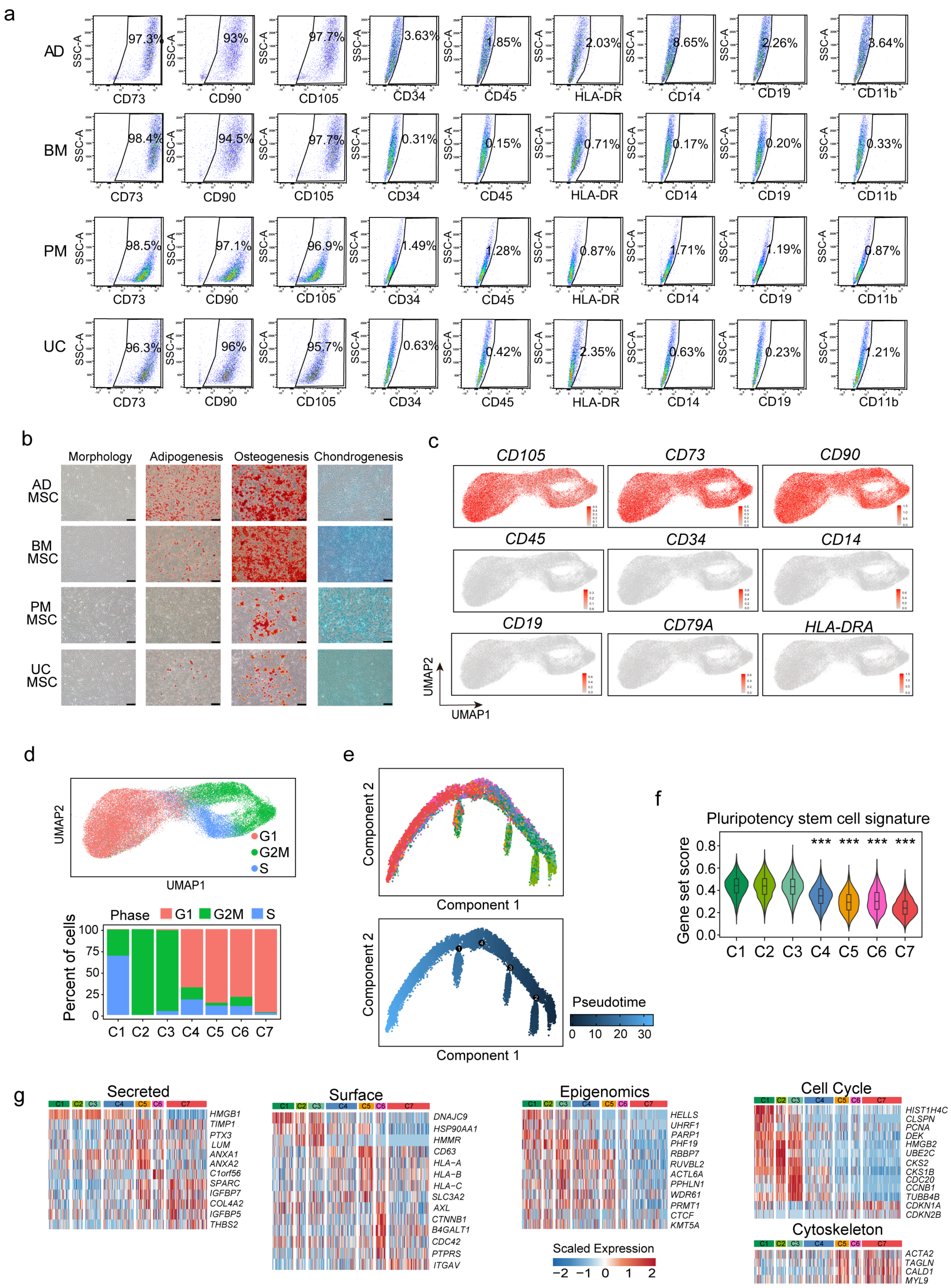

**Supplementary Fig. 1: Single-cell transcriptome analysis of human MSCs**

**(a),** Flow cytometry analysis showing that human MSC lines express CD105, CD73 and CD90 and lack expression of CD45, CD34, CD14 or CD11b, CD79a or CD19 and HLA-DR surface molecules. **(b),** Morphologic comparison of MSCs isolated from different tissue sources showing a similar culture density immediately before scRNA-seq sample preparation. Trilineage differentiation of our samples could be induced, as indicated by Oil Red-O staining (Adipogenesis), Alizarin Red S staining (Osteogenesis) and Alcian Blue staining (Chondrogenesis). Data are representative of three independent experiments. Scale bar, 200μm. **(c),** Feature plot showing expression of nine MSC surface antigen genes defined by ISCT criteria. Color represents the normalized expression values from Seurat RNA assay. The displayed values were non-batch corrected. **(d),** Cell cycle scoring assign each cell a score, based on its expression of G2/M and S phase markers, and predicted each cell a cycle phase (top). The percentage of cells assign to G2M phase, S phase and G1phase in each cluster were show in stacking histogram (bottom). (**e**), Monocle trajectories of MSCs colored by cluster identity (top) and predicted pseudotime value (bottom). **(f),** Violin plots showing average expression of pluripotency stem cell signature genes for each cluster. Box plot within each violin plot indicate median values, and the 25th to 75th percentiles. Asterisks on specific group represent there were statistical differences compared with cluster C1, C2 and C3, *p* values are generated by two-sided one-way ANOVA with Tukey's multiple comparisons test. (***$p < 2.2 \times 10^{-16}$, n=45,955 biologically independent cells) **(g),** Heatmap showing scaled expression of differentially expressed genes in the cells of each cluster, ordered by five gene categories (labels on top). Color represents the scaled expression values from Seurat RNA assay. The displayed values were non-batch corrected.

Supplementary Fig. 2

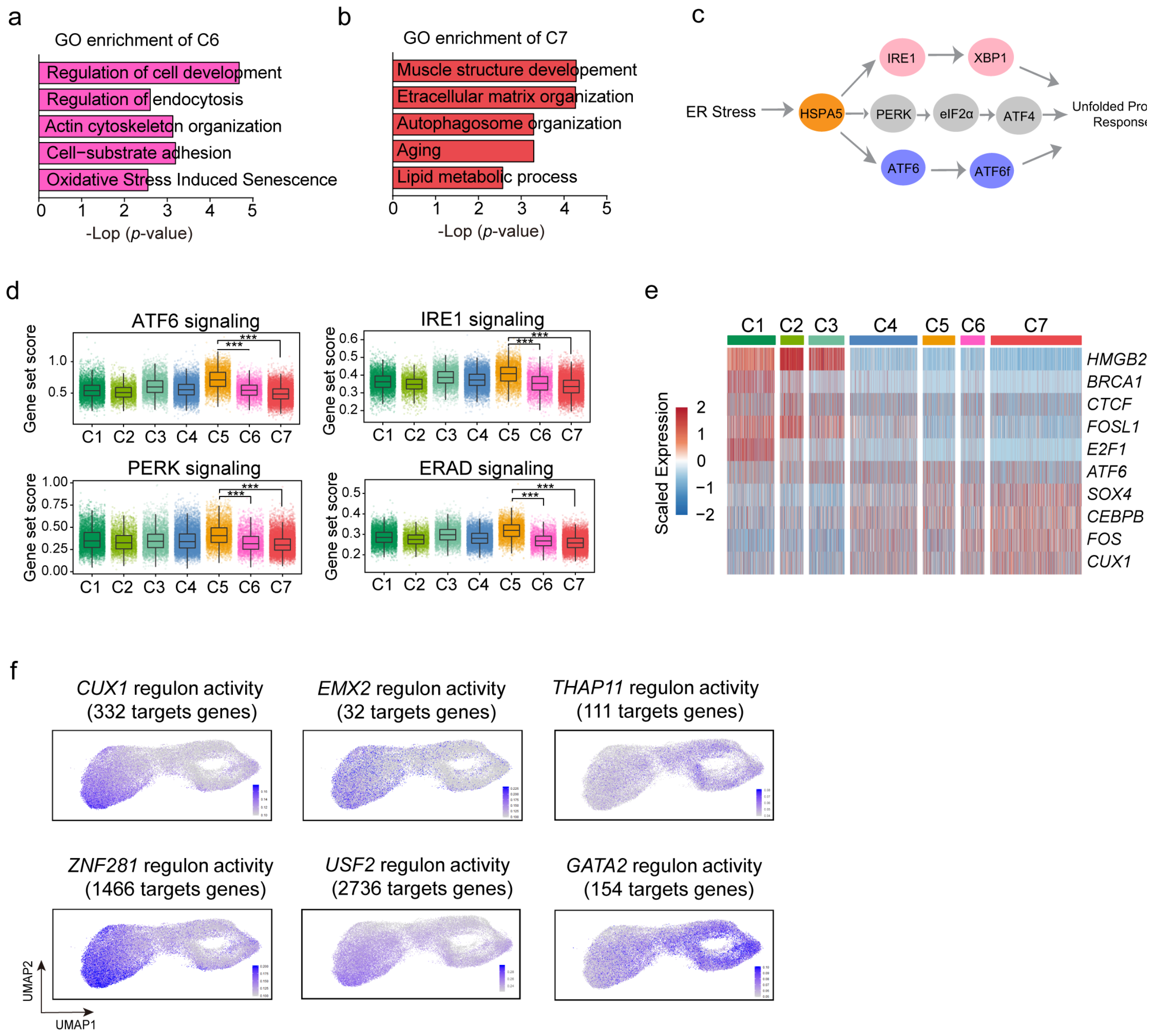


**Supplementary Fig. 2: Biochemical basis of human MSCs senescence**

**(a),** Representative Gene Ontology pathways showing enriched expression in Cluster 6. **(b),** Representative Gene Ontology pathways showing enriched expression in Cluster 7. **(c),** A schematic chart showing three main branches and corresponding principal components in the UPR pathway. **(d),** Box plots showing average expression of genes related with GO ATF6 mediated unfolded protein response, GO IRE1 mediated unfolded protein response, GO PERK mediated unfolded protein response and GO ERAD pathway (with plot center, box and whiskers corresponding to median, IQR and 1.5 × IQR, respectively). The $p$ values were determined by two-tailed Wilcoxon rank-sum test. (*** $p < 2.2 \times 10^{-16}$; n=4621 biologically independent cells in C5; n=3409 biologically independent cells in C6; n=13105 biologically independent cells in C7). **(e),** Heatmap showing expression of selected transcriptional factors related to cellular senescence from each cluster. Color represents the scaled expression values from Seurat RNA assay. The displayed values were non-batch corrected. **(f),** Feature plot showing the activity of six newly identified transcriptional regulons that may related to the senescent progress of MSC.

Supplementary Fig. 3

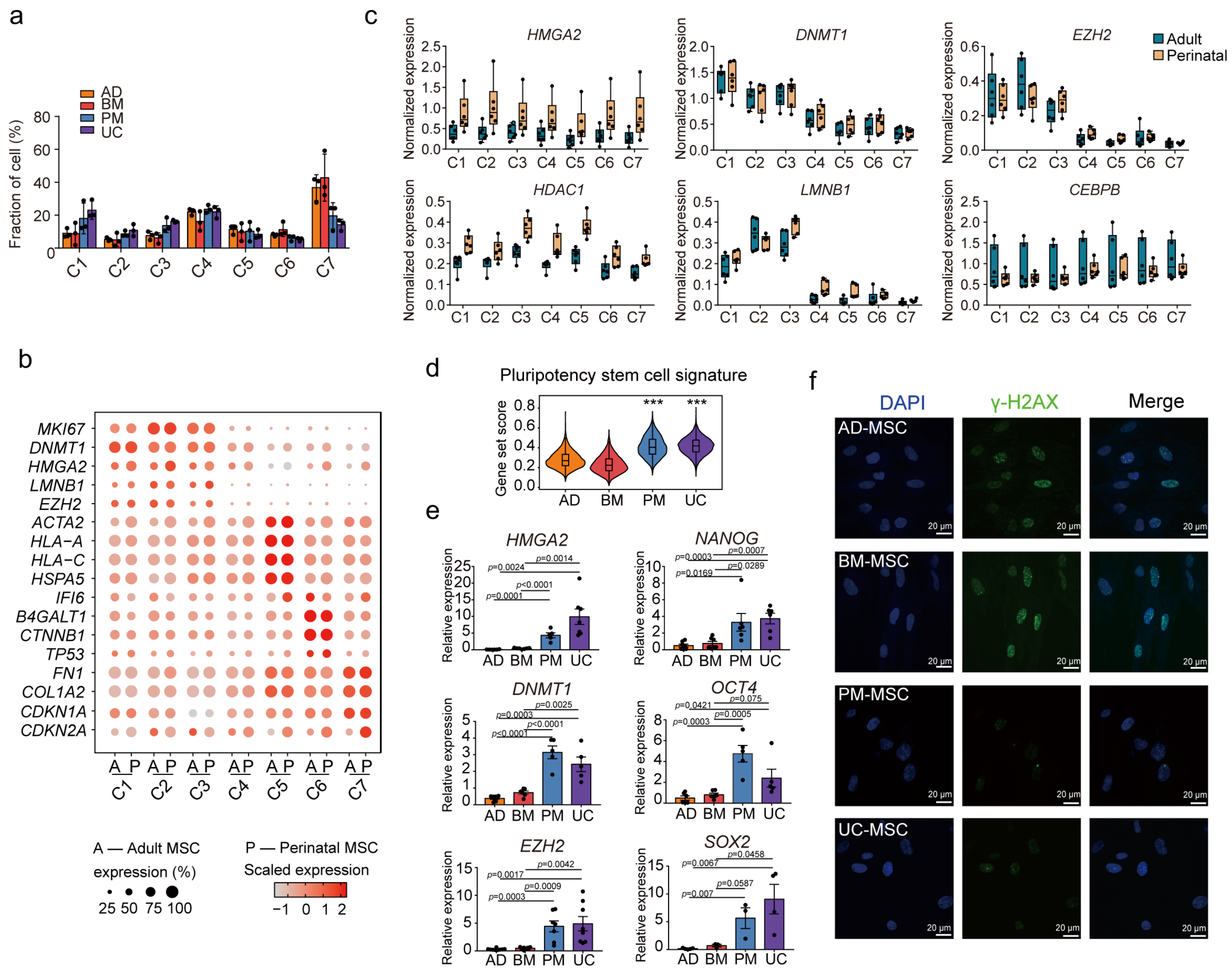

**Supplementary Fig. 3: MSCs isolated from different human tissues are heterogenous in terms of cellular senescence status**

**(a),** Fractions of MSC subpopulations in MSC scRNA samples derived from AD, BM, PM and UC. Data represent the mean ± SD (n=3 independent samples per group). **(b),** Dot plot displaying the proportion of MSC expression and the expression of signature genes for each cluster in adult and perinatal MSCs. Color represents the scaled expression values from Seurat RNA assay. The displayed values were non-batch corrected. **(c),** Box plots comparing the expression of selected feature genes in each cluster between MSCs from adult and perinatal tissue (n=6 independent samples per group). Data represents the average normalized expression values from Seurat RNA assay. The displayed values were non-batch corrected. **(d),** Violin plots of the pluripotency stem cell signature gene score of cells from each tissue origin. Asterisks on specific groups represent significant differences compared with cells derived from AD and BM. *P* values were generated by two-sided one-way ANOVA with Tukey's multiple comparisons test. (***$p < 2.2 \times 10^{-16}$, n=45,955 biologically independent cells) **(e),** Relative mRNA expression of genes related to the anti-senescence (*HMGA2*, *DNMT1*, *EZH2*) and pluripotency stem cell signature (*NANOG*, *OCT4*, *SOX2*) in AD, BM, PM and UC MSCs, as measured by real-time qPCR. Data are means ± SEM. For each gene, data are representative of 3 independent experiments (HMGA2: nAD=6, nBM=7, nPM=5, nUC=7; DNMT1: nAD=7, nBM=6, nPM=5, nUC=5; EZH2: nAD=9, nBM=8, nPM=7, nUC=8; NANOG: nAD=7, nBM=7, nPM=6, nUC=6; SOX2: nAD=5, nBM=3, nPM=3, nUC=4; OCT4: nAD=6, nBM=6, nPM=5, nUC=5). The *p* values were generated by two-tailed Student's *t*-test. **(f),** DNA damage was analyzed by immunofluorescence staining for γ-H2AX. Data represent one independent experiment. The scale bar represents 20μm. In the box plots, the plot center, box and whiskers corresponding to median, IQR and 1.5 × IQR, respectively. Source data are provided as a Source Data file.

Supplementary Fig. 4

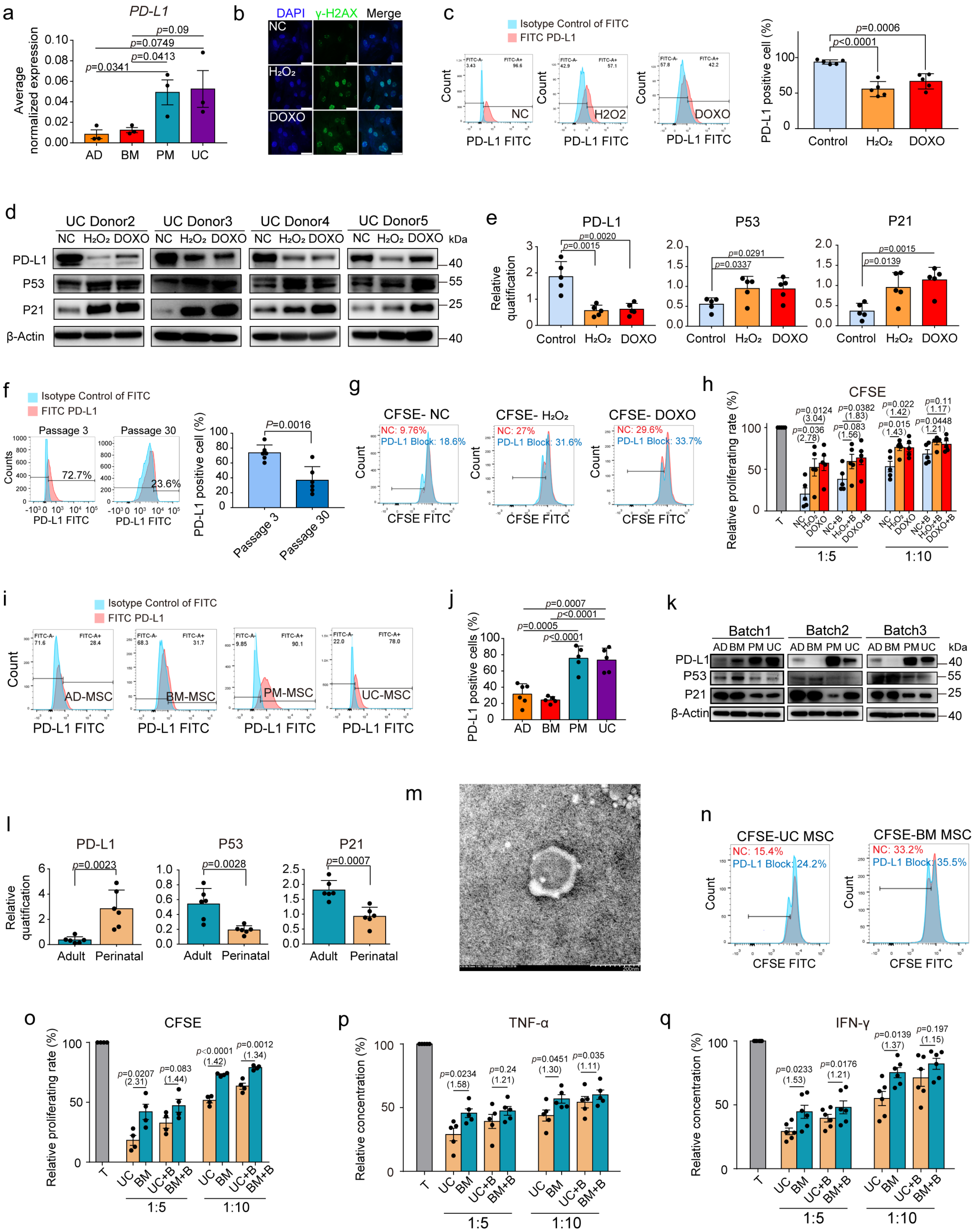

**Supplementary Fig. 4: Attenuated PD-L1 expression accounts for the impaired immunosuppressive activity of senescent MSCs**

**(a),** Bar plots shows pseudobulk normalized expression value of *PD-L1* from Seurat RNA assay (means ± SD, n=3 independent samples per group). **(b),** Immunofluorescence staining for γ-H2AX. Scale bar, 40μm. Data represent one independent experiment. **(c),** Flow cytometry examination of PD-L1 expression in UC-MSCs after $H_2O_2$ or doxorubicin treatment, represent three independent experiments (means ± SD, n=5 independent samples per group). **(d),** Western blot showing PD-L1, P53 and P21 protein expression in UC-MSCs, representative of 4 independent experiments. **(e),** Bar plots showing the relative quantification results of the protein bands in Fig. 4e and supplementary Fig. 4d. (means ± SD, n= 5 independent experiments). **(f),** PD-L1 expression in passage 3 or passage 30 UC-MSCs, as detected by flow cytometry, represent three independent experiments (means ± SD, n=6 independent samples per group). **(g),** Representative flow cytometry plots showing the ratio of CFSE dilution assays. **(h),** Bar plots showing the relative proliferation rates of $CD4^+$ T cells cocultured with normal control (NC), $H_2O_2$-treated or doxorubicin-treated MSCs (means ± SEM, n= 5 independent experiments). **(i-j),** PD-L1 expression on MSCs from adult and perinatal tissue detected by flow cytometry (means ± SEM, n=5 samples for BM, PM and UC; n=6 for AD). **(k),** Western blot showing PD-L1, P53 and P21 protein expression in adult and perinatal MSCs, pooled of 3 independent experiments. **(l),** Bar plots showing the relative quantification results of the protein bands in supplementary Fig. 4l, pooled of 3 independent experiments. (means ± SD, n=6 independent samples per group). **(m),** Electron microscopy image of collected EVs, represent one independent experiment. Scale bar, 200nm. **(n),** Representative flow cytometry plots. **(o),** Bar plots showing the relative proliferation rates of $CD4^+$ T cells cocultured with BM- or UC-MSCs (means ± SEM, n=4 independent experiments). **(p-q),** ELISA depicting TNF-α (p) and IFN-γ (q) levels in the co-culture supernatant Data represent four independent experiments (means ± SEM; n=5 independent samples per group for **p**; n=6 independent samples per group for **q**). For **g**, **h, n, o, p** and **q**, multiples of the mean values between the two groups are marked in parentheses below the *p*-value. For **e** and **l,** quantitative samples (gels/blots) derive from the same experiment were processed in parallel. All *p*-values were determined by two-tailed Student's *t*-test. Source data are provided as a Source Data file.

Supplementary Fig. 5

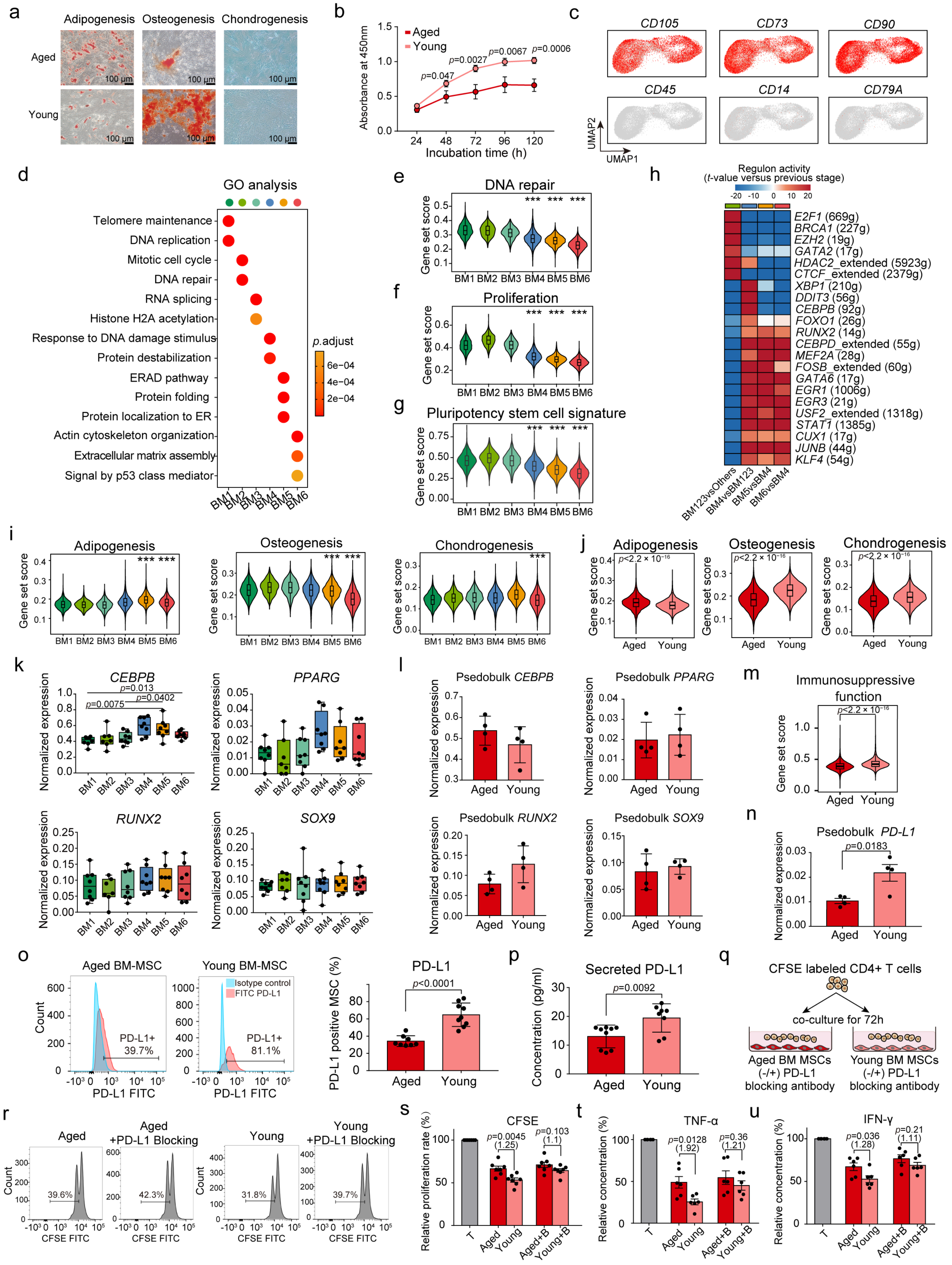

**Supplementary Fig. 5: BM-MSCs derived from aged donors possessed a more senescent phenotype and impaired immunomodulatory function**

**(a),** Trilineage differentiation of BM-MSC samples, represent three independent experiments. Scale bar, 100μm. **(b),** Proliferation of MSCs from young and aged BM donors. Data represent one independent experiment (means ± SEM; n=15 independent samples for young; n=9 independent samples for aged). **(c),** Feature plot showing log-normalized expression of MSC surface antigen genes. **(d),** GO analysis of DEGs for each subcluster in BM-MSCs. **(e-g),** Violin plots showing average expression of DNA repair genes (e), proliferation genes (f) and pluripotency stem cell signature genes (g) for each cluster. **(h),** Heatmap showing the *t*-values of regulon activity derived by the generalized linear model. **(i),** Violin plots showing average expression of genes related to adipogenesis, osteogenesis and chondrogenesis for each MSC cluster. **(j),** Violin plots showing average normalized expression of adipogenesis genes, osteogenesis genes and chondrogenesis genes of young and aged BM-MSCs (n=31,907 biologically independent cells). **(k),** Box plot showing the pseudobulk expression of *CEBPB*, *PPARG*, *RUNX2* and *SOX9* in each BM-MSC cluster (n=8 independent samples each cluster). **(l),** Pseudobulk expression of *CEBPB*, *PPARG*, *RUNX2* and *SOX9* from pseudobulk young and aged BM-MSC data (means ± SD; n=4 independent samples each group). **(m),** Violin plots showing average expression of immunosuppressive genes of young and aged BM-MSCs (n=31,907 biologically independent cells). **(n),** Bar plot showing the pseudobulk *PD-L1* expression of young and aged BM-MSCs (means ± SEM; n=4 independent samples each group). **(o),** The proportion of PD-L1-positive cells calculated by comparison with the FITC isotype control (means ± SD; n=8 independent samples for aged; n=9 independent samples for young). (**p),** ELISA depicting the PD-L1 concentration in the culture supernatant of BM-MSCs. Data represent 2 independent experiments (means ± SD; n=9 for aged, n=8 for young). **(q),** Experimental design. **(r),** Representative flow cytometry plots. **(s),** Bar plots showing the relative proliferation rates of $CD4^+$ T cells cocultured with young or aged BM-MSCs. Data represent 3 independent experiments (means ± SEM; n=7 independent samples per group). **(t-u),** ELISA depicting TNF-α (t) and IFN-γ (u) levels in the co-culture supernatant, represent 3 independent experiments (means ± SEM; n=6 independent samples per group). All box plots indicate median values, and the 25th to 75th percentiles. For **s-u**, $CD4^+$ T cells cocultured with MSCs for 72 h in the presence of isotype or PD-L1 blocking antibody (+B), multiples of the mean values between the two groups are marked in parentheses below the *p*-value. For **b**, **j**, **k**, **m**, **n**, **o**, **p**, **s**, **t**, and **u,** *p* values were determined by two-tailed unpaired Student's *t*-test. For **e-g** and **i,** *p* values were generated by two-sided one-way ANOVA with Tukey's multiple

comparisons test, asterisks on specific group represent there were statistical differences compared with cluster BM1, BM2 and BM3. (***$p < 2.2 \times 10^{-16}$; n=31,907 biologically independent cells). Source data are provided as a Source Data file.

Supplementary Fig. 6

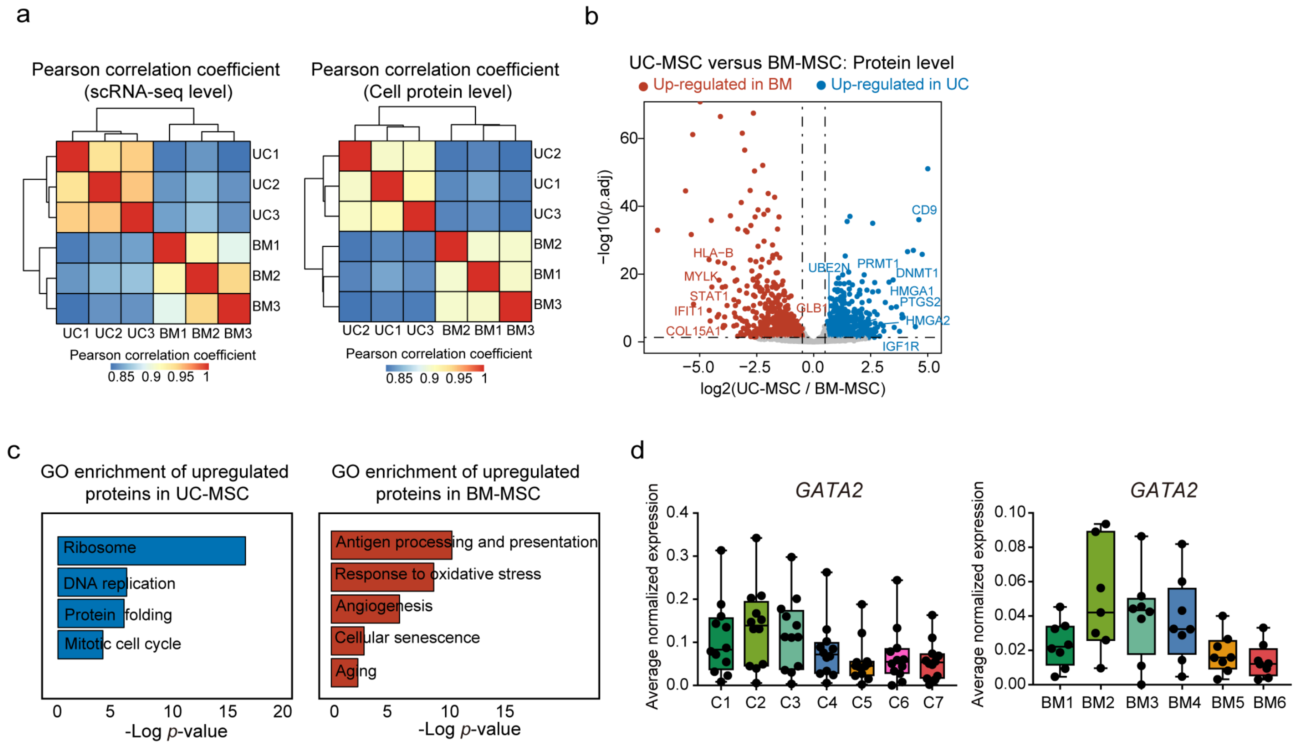


**Supplementary Fig. 6: GATA2 was identified as a crucial modulator of MSC senescence**

**(a),** Heatmaps showing the Pearson correlation coefficients of the 6 MSC samples from BM and UC at the scRNA level (left) and cell proteome level (right). **(b),** Volcano plots showing the differentially expressed proteins calculated by DESeq2. A log-transformed fold change value greater than 0.5 and adjusted *p* values less than 0.05 were used to define significantly upregulated proteins; the selected differentially expressed proteins are labeled. **(c),** Bar plot representing enriched GO pathways of the upregulated proteins (log2fold change>1; *p*.adj<0.05) in UC-MSCs and BM-MSCs. **(d),** (left) Box plot showing the average expression of *GATA2* in MSC from adult and perinatal tissue (n=12 independent pseudobulk samples per cluster); (right) and the gradually reduced expression of *GATA2* during aging process is showed in BM-MSCs derived from young and aged donors (n=8 independent pseudobulk samples per cluster). Each dot represents the average normalized expression value of *GATA2* from Seurat RNA assay. The displayed values were non-batch corrected. The box plot center and box correspond to the median and the 25th to 75th percentiles, respectively. Source data are provided as a Source Data file.

## Supplementary Fig. 7

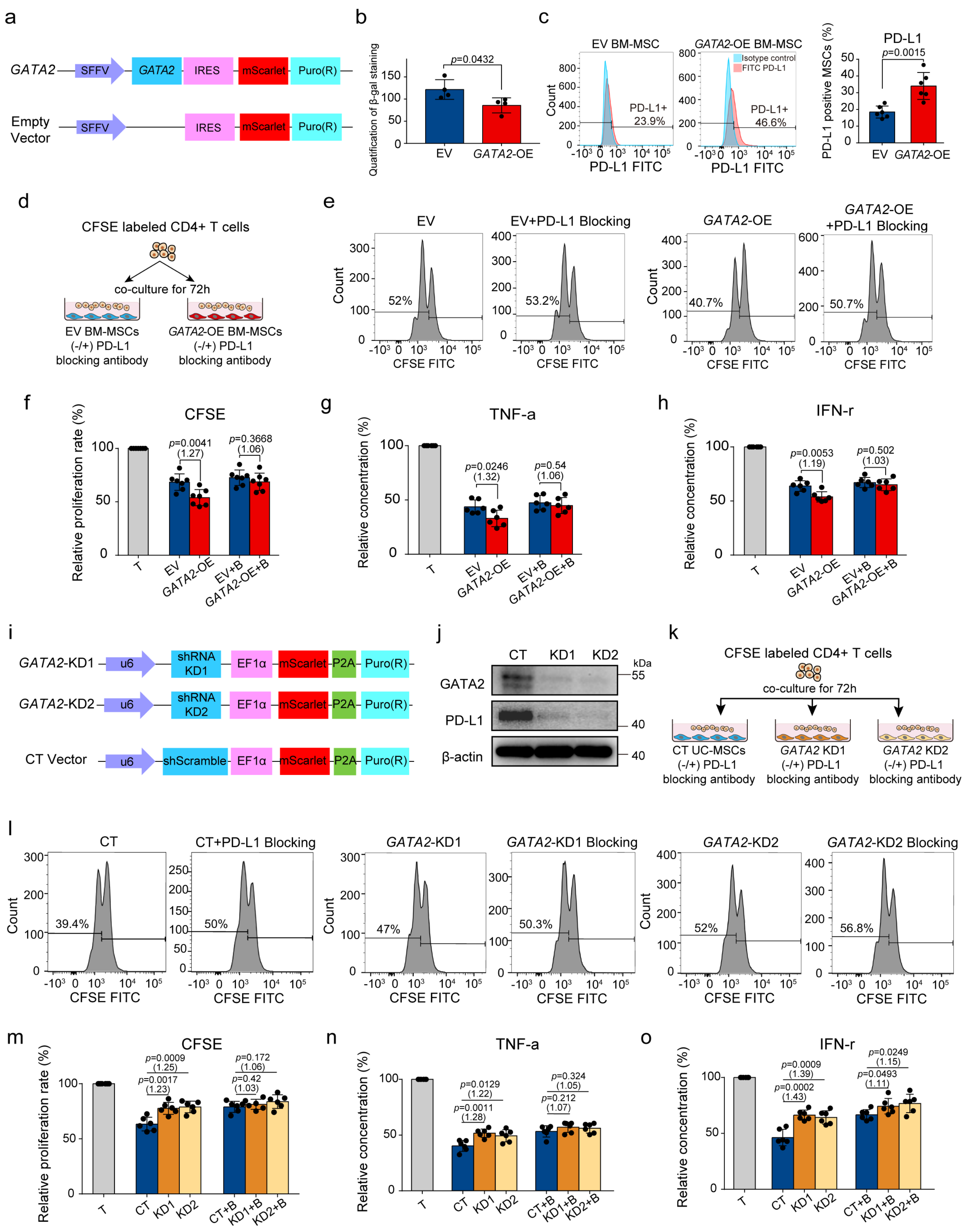

**Supplementary Fig. 7: GATA2 enhance the immunomodulatory ability of MSC through the upregulation of PD-L1**

**(a),** Schematic representation of the plasmid structure for *GATA2* overexpression. **(b),** The quantitative data of SA-β-gal-positive BM-MSCs of passage 15 are shown in the bar plot. Data are representative of 3 independent experiments (means ± SD, n=4 independent samples per group). **(c),** (left) Representative flow cytometry plots. (right) Bar plot showing PD-L1 expression on EV or *GATA2*-OE MSCs by flow cytometry, represent 3 independent experiments (means ± SD, n=6 independent samples per group). **(d),** Experimental design. **(e),** Representative flow cytometry plots showing the proportion of daughter $CD4^+$ T cells. **(f),** Bar plots showing the relative proliferation rates of $CD4^+$ T cells cocultured with CT or *GATA2*-KD MSCs, represent 3 independent experiments (means ± SD, n=7 independent samples per group). **(g-h),** ELISA detecting TNF-α (g) and IFN-γ (h) protein levels in coculture supernatant secreted by T cells after coculture with CT or *GATA2*-KD MSCs. Data are representative of 2 independent experiments (means ± SD, n=6 independent samples per group). **(i),** Schematic representation of the plasmid structure for *GATA2* knockdown. **(j),** Western blot analysis of GATA2 and PD-L1 protein in control and *GATA2*-KD UC-MSCs. Data represent one independent experiment. **(k),** Experimental design. **(l),** Representative flow cytometry plots showing the proportion of daughter $CD4^+$ T cells. **(m),** Bar plots showing the relative proliferation rates of $CD4^+$ T cells cocultured with CT or *GATA2*-KD MSCs Data represent 3 independent experiments (means ± SD, n=6 independent samples per group). **(n-o),** ELISA detecting TNF-α (n) and IFN-γ (o) protein levels in cell culture supernatant secreted by T cells after coculture with CT or *GATA2*-KD MSCs, representative of 2 independent experiments (means ± SD, n=6 independent samples per group). For **f**, **g**, **h**, **m**, **n** and **o,** T cells were co-cultured with MSCs for 72h in the presence of isotype or PD-L1 blocking antibody (+B), multiples of the mean values between the two groups are marked in parentheses below the *p*-value. All *p*-values were generated by two-tailed unpaired Student's *t*-test. Source data are provided as a Source Data file.

Supplementary Fig. 8

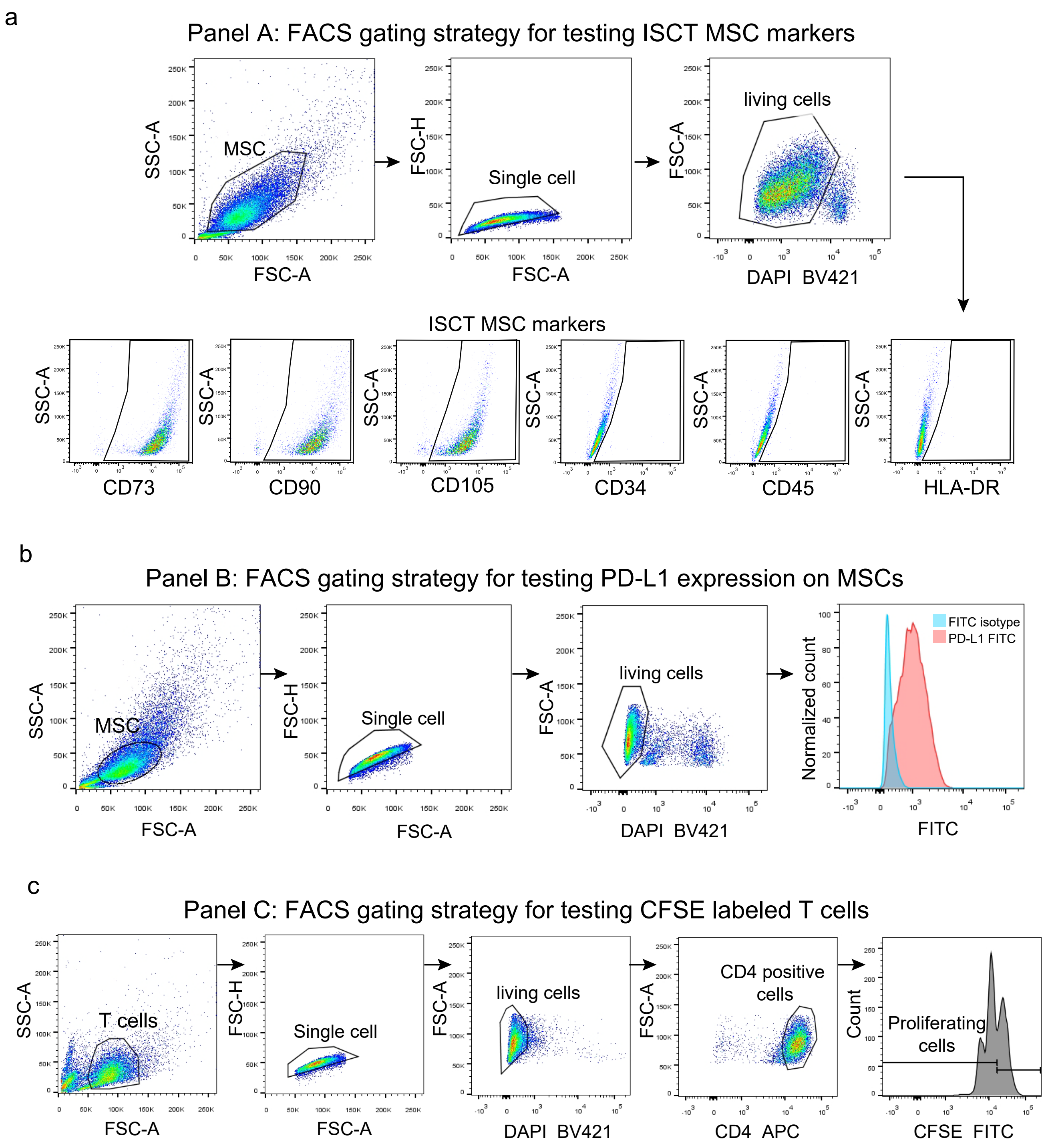


**Supplementary Fig. 8: Gating strategy for FACS analysis**

**(a),** Schematic representation of FACS sequential gating strategies for testing ISCT MSC markers. **(b),** Schematic representation of FACS sequential gating strategies for testing PD-L1 expression on MSCs. **(c),** Schematic representation of FACS sequential gating strategies for testing CFSE labeled T cells.
