# Supplementary material for: Multi-omics analysis of human mesenchymal stem cells shows cell aging that alters immunomodulatory activity through the downregulation of PD-L1
Source: Nat Commun. 2023 Jul 20;14:4373. doi: 10.1038/s41467-023-39958-5 (PMC10359415; doi:10.1038/s41467-023-39958-5)
Supplement: Supplementary file 3 — Description of Additional Supplementary Files [file 41467_2023_39958_MOESM3_ESM.pdf]

Description of Additional Supplementary Files

Name: Supplementary Data 1
Description: Basic information for MSCs sample donors and scRNA-seq analysis.

Name: Supplementary Data 2
Description: Pseudobulk DEGs of each MSC cluster.

Name: Supplementary Data 3
Description: Lists of genes used in gene set analysis

Name: Supplementary Data 4
Description: Results for GSVA analysis

Name: Supplementary Data 5
Description: Regulon targets information for SCENIC analysis

Name: Supplementary Data 6
Description: Results of generalized linear model (GLM) analysis

Name: Supplementary Data 7
Description: Differentially expressed Proteins on MSC EVs Proteome level.

Name: Supplementary Data 8
Description: Pseudobulk DEGs of each young and aged BM-MSC cluster.

Name: Supplementary Data 9
Description: Results of generalized linear model (GLM) analysis in Fig. S5h

Name: Supplementary Data 10
Description: Differentially expressed Proteins or Genes on MSC Proteome level or pseudobulk scRNA level.

Name: Supplementary Data 11
Description: Results of 2D enrichment analysis.

Name: Supplementary Data 12
Description: Supporting information for Fig. 6e.

Name: Supplementary Data 13
Description: Sequences of primers and shRNA.
