# Supplementary material for: Multi-omics analysis of human mesenchymal stem cells shows cell aging that alters immunomodulatory activity through the downregulation of PD-L1
Source: Nat Commun. 2023 Jul 20;14:4373. doi: 10.1038/s41467-023-39958-5 (PMC10359415; doi:10.1038/s41467-023-39958-5)
Supplement: Supplementary file 5 — Reporting Summary [file 41467_2023_39958_MOESM5_ESM.pdf]

nature portfolio

Corresponding author(s): Lei Zhang, Xiaomin Zhang, Jun Wei

Last updated by author(s): Jun 30, 2023

Reporting Summary

Nature Portfolio wishes to improve the reproducibility of the work that we publish. This form provides structure for consistency and transparency in reporting. For further information on Nature Portfolio policies, see our Editorial Policies and the Editorial Policy Checklist.

Statistics

For all statistical analyses, confirm that the following items are present in the figure legend, table legend, main text, or Methods section.

n/a	Confirmed	
☐	☒	The exact sample size (*n*) for each experimental group/condition, given as a discrete number and unit of measurement
☐	☒	A statement on whether measurements were taken from distinct samples or whether the same sample was measured repeatedly
☐	☒	The statistical test(s) used AND whether they are one- or two-sided<br>*Only common tests should be described solely by name; describe more complex techniques in the Methods section.*
☐	☒	A description of all covariates tested
☐	☒	A description of any assumptions or corrections, such as tests of normality and adjustment for multiple comparisons
☐	☒	A full description of the statistical parameters including central tendency (e.g. means) or other basic estimates (e.g. regression coefficient) AND variation (e.g. standard deviation) or associated estimates of uncertainty (e.g. confidence intervals)
☐	☒	For null hypothesis testing, the test statistic (e.g. *F*, *t*, *r*) with confidence intervals, effect sizes, degrees of freedom and *P* value noted<br>*Give P values as exact values whenever suitable.*
☒	☐	For Bayesian analysis, information on the choice of priors and Markov chain Monte Carlo settings
☒	☐	For hierarchical and complex designs, identification of the appropriate level for tests and full reporting of outcomes
☐	☒	Estimates of effect sizes (e.g. Cohen's *d*, Pearson's *r*), indicating how they were calculated

Our web collection on statistics for biologists contains articles on many of the points above.

Software and code

Policy information about availability of computer code

Data collection	bcl2fastq (version 2.1.9),BD FACSDIva (version 6.0), R (version 3.6; version 4.2.1), Python (version 2.7.5), SWATH Variable Window Calculator (software version 1.1), ProteinPilot software (version 2.0) and SWATH software (version 2.0) were included for data collection in this research.
Data analysis	The FlowJo (version 10.0.7), GraphPad Prism (version 7.00), R package Seurat (version 3.1.0; version 4.2.0), CellRanger (version 2.2.0; version 5.0.1), Monocle(version 2.26.0), GSEA software (version 4.0.3), SCENIC R-package (SCENIC version 1.1.3; RcisTarget version 1.6.0; AUCell version 1.8.0; with RcisTarget hg19 motif databases), GENIE3 package (version 1.8.0), GSVA package (version 1.36.2), Python Scanpy package (version 1.8.2), Velocyto.py (version 0.17.17), Velocyto. R package (version 0.6), DESeq2 (version 1.24.0), limma (version 3.40.6), fastq-dump (version 2.5.7), Trimgalore (version 0.6.6), Bowtie2 (version 2.3.5.1), MACS2 (version 2.2.7.1), deepTools (version 3.5.1), Integrative Genomics Viewer136 (IGV, version 2.4.16), Perseus software (version 1.6.14.0), Nikon NIS Element software (version 5.21.00), ImageJ (version 1.52p), Metascape (version v3.5.20211218; http://metascape.org/gp/index.html), ggplot2 R package (version 3.2.1) and pheatmap R package (version 1.0.12) were included for data analysis in this research.<br><br>Codes related to data screening and major analysis are deposited in the GitHub repository (https://github.com/GaoYuchenPUMC/MSC_paper, https://zenodo.org/record/8026091).

For manuscripts utilizing custom algorithms or software that are central to the research but not yet described in published literature, software must be made available to editors and reviewers. We strongly encourage code deposition in a community repository (e.g. GitHub). See the Nature Portfolio guidelines for submitting code & software for further information.

Data

Policy information about availability of data

All manuscripts must include a data availability statement. This statement should provide the following information, where applicable:

- Accession codes, unique identifiers, or web links for publicly available datasets
- A description of any restrictions on data availability
- For clinical datasets or third party data, please ensure that the statement adheres to our policy

The scRNA-seq data and read counts data of MSCs were deposited at the National Center for Biotechnology Information's Gene Expression Omnibus with accession number: GSE200161. The raw scRNA sequence data of young and aged BM-MSC have been deposited in the Genome Sequence Archive in National Genomics Data Center, China National Center for Bioinformation / Beijing Institute of Genomics, Chinese Academy of Sciences (GSA-Human: HRA003258) that are publicly accessible at (https://ngdc.cncb.ac.cn/gsa-human/browse/HRA003258). The mass spectrometry proteomics data of MSCs at both the cellular (dataset identifier: PXD033812) and extracellular vesicle (dataset identifier: PXD042977) levels have been deposited to the ProteomeXchange Consortium (http://proteomecentral.proteomexchange.org). The RDS files, including meta data, labeled assays, and reduction map information have been made publicly available via Zenodo (https://zenodo.org/record/8026174) in order to ensure the reproducibility of the scRNA-seq data. The GRCh38 reference genome used to map the single-cell RNA-seq data and ChIP-seq data was downloaded from the 10× genomics website (http://cf.10xgenomics.com/supp/cell-exp/refdata-cellranger-GRCh38-1.2.0.tar.gz). The publicly available auxiliary input databases for SCENIC analysis were downloaded from the cisTarget resources website (https://resources.aertslab.org/cistarget/). The MS/MS data were searched against the human UniProt database (https://www.uniprot.org/). Source data are provided with this paper.

Human research participants

Policy information about studies involving human research participants and Sex and Gender in Research.

Reporting on sex and gender	The biological sex of sample donors was considered into study design, but our findings were not only apply to one sex. The sex of sample donors was determined based on self-reporting and disaggregated sex data was provided in the Supplementary Data 1. No sex or gender based analyses were performed due to insufficient sample size.
Population characteristics	Population characteristics are outlined in Supplementary Data 1.
Recruitment	For MSC donor, only healthy individuals were recruited to exclude the impact of any drug treatment or chronic condition on the immune status of the patients, all participants were tested negative for HCV, HBV and HIV. Participants of both biological sex were recruited to avoid misleading conclusions that could refer only to one sex. All the donors or their guardians were provided written informed consent for sample collection and data analysis, and we have obtained consent from donors to publish identifying information, including the biological sex and age information. BM-MSCs were obtained from bone marrow aspirates through iliac bone puncture, AD-MSCs are acquired through liposuction surgery, PM- and UC-MSCs are obtained from neonatals following eutocia or normal caesarean section.<br>PBMC samples were collected via venous blood collection from the voluntary blood donation center at the Institute of Hematology and Hospital of Blood Disease, Chinese Academy of Medical Sciences. Voluntary donors were excluded if they were undergoing drug treatment, had chronic conditions, or had infectious diseases that could impact their immune status. All donors signed a consent form agreeing to sample collection and the use of samples for scientific research.
Ethics oversight	This study was approved by the Ethics Committee of the State Key Laboratory of Experimental Hematology, Institute of Hematology and Hospital of Blood Disease, Chinese Academy of Medical Sciences & Peking Union Medical College, Tianjin, China. All the people in this study provided written informed consent for sample collection and data analyses.

Note that full information on the approval of the study protocol must also be provided in the manuscript.

Field-specific reporting

Please select the one below that is the best fit for your research. If you are not sure, read the appropriate sections before making your selection.

☒ Life sciences ☐ Behavioural & social sciences ☐ Ecological, evolutionary & environmental sciences

For a reference copy of the document with all sections, see nature.com/documents/nr-reporting-summary-flat.pdf

Life sciences study design

All studies must disclose on these points even when the disclosure is negative.

Sample size	No statistical tests were performed for sample size calculation. Sample sizes for scRNA-seq, LC-MS/MS and in vitro experiments were determined based on sample availability, the number of experimental or control groups required to draw meaningful conclusions. Based on references to previous experience and published research in our laboratory (Stem Cell Res Ther. 2016 Apr 4;7:49.; Haematologica. 2020 Mar;105(3):661-673.), the sample sizes were considered sufficient for statistical analysis and to generate significant results.
Data exclusions	No data was excluded.

Replication	Each experiment in this study was independently repeated for at least 3 times to generate final conclusion. The replication of experiments showed similar results or trend.
Randomization	During the in vitro experimental process, MSCs were randomly allocated to the experimental and control groups. Randomization is not relevant to other experiments.
Blinding	Blinding was implemented during the library preparation and data sequencing stage of bioinformatic data. The operators involved in the single-cell transcriptome library preparation and sequencing process, as well as the operators involved in protein sequencing by mass spectrometry, were provided with sample numbers but were not provided with specific information regarding sample identity or grouping. The bioinformatic analysis was not performed in blind, since data analysis required specific information regarding sample collection and grouping.<br>The investigators were not blinded to sample collection stage and sample allocation during experiments, because the information of the donor age, sample tissue origin and different treatment among groups was essential and correctly to conduct the studies.

Reporting for specific materials, systems and methods

We require information from authors about some types of materials, experimental systems and methods used in many studies. Here, indicate whether each material, system or method listed is relevant to your study. If you are not sure if a list item applies to your research, read the appropriate section before selecting a response.

Materials & experimental systems

n/a	Involved in the study
☐	☒ Antibodies
☐	☒ Eukaryotic cell lines
☒	☐ Palaeontology and archaeology
☒	☐ Animals and other organisms
☒	☐ Clinical data
☒	☐ Dual use research of concern

Methods

n/a	Involved in the study
☒	☐ ChIP-seq
☐	☒ Flow cytometry
☒	☐ MRI-based neuroimaging

Antibodies

Antibodies used	1. CD11b-APC (561015, BD Biosciences, 1:100 dilution)<br>2. CD14-APC (555399, BD Biosciences, 1:100 dilution)<br>3. CD19-APC (555415, BD Biosciences, 1:100 dilution)<br>4. CD34-APC (560940, BD Biosciences, 1:100 dilution)<br>5. CD45-APC (560915, BD Biosciences, 1:100 dilution)<br>6. HLA-DR-FITC (555560, BD Biosciences, 1:100 dilution)<br>7. CD73- PerCP/Cyanine5.5 (344013, Biolegend, 1:100 dilution)<br>8. CD90-APC (559869, BD Biosciences, 1:100 dilution)<br>9. CD105-PE (560839, BD Biosciences, 1:100 dilution)<br>10. PD-L1-FITC (393606, Biolegend, 1:50 dilution)<br>11. CD4-APC (317416, Biolegend, 1:50 dilution)<br>12. FITC Isotype (555748, BD Biosciences, 1:100 dilution)<br>13. Purified anti-human CD274 (B7-H1, PD-L1) Antibody (329716, Biolegend, 1:400 dilution, working concentration: 5 μg/mL)<br>14. PDL1 (ab213524, Abcam, 1:1000 dilution)<br>15. TP53 (2527S, Cell Signaling Technology, 1:1000 dilution)<br>16. P21 (ab109520, Abcam, 1:1000 dilution)<br>17. β-Actin (abs137975, Absin Bioscience Inc, 1:1000 dilution)<br>18. HRP-conjugated secondary Goat Anti-Mouse IgG (H+L) antibody (115-035-003, AffiniPure, 1:1000 dilution)<br>19. HRP-conjugated secondary Goat Anti-Rabbit IgG (H+L) antibody (111-035-003, AffiniPure, 1:1000 dilution)<br>20. anti-γ-H2AX antibody (80312S, Cell signaling technology, 1:100 dilution)<br>21. Alexa Fluor goat-anti-mouse IgG (H+L) antibody (A11001, Invitrogen,1:100 dilution)<br>22. GATA2 (ab109241, Abcam, 1:1000 dilution for WB; 1:50 dilution for ChIP-PCR)
Validation	All antibodies used in this study were obtained from commercial source, and were validated by the manufacturer as follows:<br>1. CD11b-APC, CAT#561015, BD Biosciences. The manufacturer states that this antibody can be used for Flow cytometry. Reactivity: Human (QC Testing); Isotype: Mouse IgG1. (https://www.bdbiosciences.com/en-nz/products/reagents/flow-cytometry-reagents/research-reagents/single-color-antibodies-ruo/apc-mouse-anti-human-cd11b.561015)<br>2. CD14-APC, CAT#555399, BD Biosciences. The manufacturer states that this antibody can be used for Flow cytometry. Reactivity: Human (QC Testing), Rhesus, Cynomolgus, Baboon, Dog (Tested in Development); Isotype:Mouse IgG2a. (https://www.bdbiosciences.com/en-nz/products/reagents/flow-cytometry-reagents/research-reagents/single-color-antibodies-ruo/apc-mouse-anti-human-cd14.555399)<br>3. CD19-APC, CAT#555415, BD Biosciences. The manufacturer states that this antibody can be used for Flow cytometry. Reactivity: Human (QC Testing); Isotype: Mouse IgG1. (https://www.bdbiosciences.com/en-nz/products/reagents/flow-cytometry-reagents/research-reagents/single-color-antibodies-ruo/apc-mouse-anti-human-cd19.555415)

4. CD34-APC, CAT#560940, BD Biosciences. The manufacturer states that this antibody can be used for Flow cytometry. Reactivity: Human (QC Testing); Isotype: Mouse IgG1. (https://www.bdbiosciences.com/en-nz/products/reagents/flow-cytometry-reagents/research-reagents/single-color-antibodies-ruo/apc-mouse-anti-human-cd34.560940)
5. CD45-APC, CAT#560915, BD Biosciences. The manufacturer states that this antibody can be used for Flow cytometry. Reactivity: Human (QC Testing); Isotype: Mouse IgG1. (https://www.bdbiosciences.com/en-nz/products/reagents/flow-cytometry-reagents/research-reagents/single-color-antibodies-ruo/pe-cy-7-mouse-anti-human-cd45.560915)
6. HLA-DR-FITC, CAT#555560, BD Biosciences. The manufacturer states that this antibody can be used for Flow cytometry. Reactivity: Human (QC Testing), Rhesus, Cynomolgus, Baboon, Dog, Rabbit (Tested in Development); Isotype: Mouse IgG2b. (https://www.bdbiosciences.com/en-nz/products/reagents/flow-cytometry-reagents/research-reagents/single-color-antibodies-ruo/fitc-mouse-anti-human-hla-dr.555560)
7. CD73- PerCP/Cyanine5.5, CAT#344013, Biolegend. The manufacturer states that this antibody can be used for Flow cytometry. Isotype Control: PerCP/Cyanine5.5 Mouse IgG1; Verified Reactivity:Human. (https://www.biolegend.com/en-us/products/percp-cyanine5-5-anti-human-cd73-ecto-5-nucleotidase-antibody-8326)
8. CD90-APC, CAT#559869, BD Biosciences. The manufacturer states that this antibody can be used for Flow cytometry. Reactivity: Human (QC Testing), Rhesus, Cynomolgus, Baboon, Pig, Dog (Tested in Development); Isotype: Mouse BALB/c IgG1. (https://www.bdbiosciences.com/en-us/products/reagents/flow-cytometry-reagents/research-reagents/single-color-antibodies-ruo/apc-mouse-anti-human-cd90.559869)
9. CD105-PE, CAT#560839, BD Biosciences. The manufacturer states that this antibody can be used for Flow cytometry. Reactivity: Human (QC Testing). (https://www.bdbiosciences.com/en-us/products/reagents/flow-cytometry-reagents/research-reagents/single-color-antibodies-ruo/pe-mouse-anti-human-cd105.560839)
10. PD-L1-FITC, CAT#393606, Biolegend. The manufacturer states that this antibody can be used for Flow cytometry. Verified Reactivity: Human; Host Species: Mouse. (https://www.biolegend.com/en-us/products/fitc-anti-human-cd274-b7-h1-pd-l1-antibody-16037)
11. CD4-APC, CAT#317416, Biolegend. The manufacturer states that this antibody can be used for Flow cytometry. Verified Reactivity: Human; Host Species: Mouse. (https://www.biolegend.com/en-us/products/apc-anti-human-cd4-antibody-3657)
12. FITC Isotype, CAT#555748, BD Biosciences. The manufacturer states that this antibody can be used for Flow cytometry for Isotype control (Routinely Tested). Reactivity: Human; Host Species: Mouse. (https://www.bdbiosciences.com/en-us/products/reagents/flow-cytometry-reagents/research-reagents/single-color-antibodies-ruo/fitc-mouse-igg1-isotype-control.555748)
13. Purified anti-human CD274 (B7-H1, PD-L1) Antibody, CAT#329716, Biolegend. The manufacturer states that this antibody can be used for Flow cytometry, IHC and Block. Verified Reactivity: Human; Host Species: Mouse. (https://www.biolegend.com/en-us/products/ultra-leaf-purified-anti-human-cd274-b7-h1-pd-l1-antibody-7746)
14. Recombinant Anti-PD-L1 antibody , CAT#ab213524, Abcam. The manufacturer states that this antibody can be used for Flow cytometry, WB, IHC-P, ICC/IF and IP. This antibody is Knockout validated. Reacts with: Human; Host species: Rabbit. (https://www.abcam.com/products/primary-antibodies/pd-l1-antibody-epr19759-ab213524.html)
15. TP53, p53 (7F5) Rabbit mAb, CAT#2527S, Cell Signaling Technology. The manufacturer states that this antibody can be used for WB, IHC, IF and ChIP. Reacts with: Human; Host species: Rabbit. (https://www.cellsignal.com/products/primary-antibodies/p53-7f5-rabbit-mab/2527?site-search-type=Products&N=4294956287&Ntt=2527s&fromPage=plp&_requestid=407572)
16. Recombinant Anti-p21 antibody, CAT#ab109520, Abcam. The manufacturer states that this antibody can be used for Flow cytometry, WB, IHC-P, ICC/IF and IP. This antibody is Knockout validated. Reacts with: Human; Host species: Rabbit. (https://www.abcam.com/products/primary-antibodies/p21-antibody-epr362-ab109520.html)
17. Beta-Actin Mouse Monoclonal Antibody, CAT#abs137975, Absin Bioscience Inc. The manufacturer states that this antibody can be used for WB, IHC, IF/ICC, ELISA. Reacts with: Human; Host species: Mouse. (https://www.absinbio.com/antibodies/loading-controls/beta-actin/beta-actin-mouse-monoclonal-antibody.html)
18. HRP-conjugated secondary Goat Anti-Mouse IgG (H+L) antibody, CAT#115-035-003, AffiniPure. The manufacturer states that this antibody is suitable for the majority of immunodetection procedures. Target: Mouse; Host: Goat. (https://www.jacksonimmuno.com/catalog/products/115-035-003)
19. HRP-conjugated secondary Goat Anti-Rabbit IgG (H+L) antibody, CAT#111-035-003, AffiniPure. The manufacturer states that this antibody is suitable for the majority of immunodetection procedures. Target: Rabbit; Host: Goat. (https://www.jacksonimmuno.com/catalog/products/111-035-003)
20. Phospho-Histone H2A.X (Ser139) (D7T2V) Mouse mAb, CAT#80312S, Cell signaling technology. The manufacturer states that this antibody can be used for WB, IHC and IF. Reacts with: Human; Host species: Mouse. (https://www.cellsignal.com/products/primary-antibodies/phospho-histone-h2a-x-ser139-d7t2v-mouse-mab/80312)
21. Alexa Fluor goat-anti-mouse IgG (H+L) antibody, CAT#A11001, Invitrogen. The manufacturer states that this antibody can be used for IHC and ICC/IF, suitable for the majority of immunodetection procedures. Target: Mouse; Host: Goat. (https://www.thermofisher.cn/cn/zh/antibody/product/Goat-anti-Mouse-IgG-H-L-Cross-Adsorbed-Secondary-Antibody-Polyclonal/A-11001)
22. Recombinant Anti-GATA2 antibody, CAT#ab109241, Abcam. The manufacturer states that this antibody can be used for ChIP, WB, and IP. This antibody is Knockout validated. Reacts with: Human; Host species: Rabbit. (https://www.abcam.com/products/primary-antibodies/gata2-antibody-epr28222-ab109241.html)

Eukaryotic cell lines

Policy information about cell lines and Sex and Gender in Research

Cell line source(s)	HEK293T cell line was obtained from Cell Center of Institute of Hematology and Blood Diseases Hospital, Chinese Academy of Medical Sciences; original source: the American Type Culture Collection (ATCC).
Authentication	HEK293T cell line was authenticated by short tandem repeat (STR) DNA profiling analysis.
Mycoplasma contamination	The 293T cell line was tested negative for mycoplasma contamination.
Commonly misidentified lines (See ICLAC register)	No commonly misidentified line was involved in this study.

Flow Cytometry

Plots

Confirm that:

- [x] The axis labels state the marker and fluorochrome used (e.g. CD4-FITC).
- [x] The axis scales are clearly visible. Include numbers along axes only for bottom left plot of group (a 'group' is an analysis of identical markers).
- [x] All plots are contour plots with outliers or pseudocolor plots.
- [x] A numerical value for number of cells or percentage (with statistics) is provided.

Methodology

Sample preparation

Peripheral blood from healthy donors was collected into EDTA anticoagulant tubes. The Ethics Committee of Tianjin Blood Disease Hospital approved the study protocol, and the donors provided written informed consent for sample collection and data analysis. Mononuclear cells were isolated by density gradient centrifugation at 700 g for 15 minutes via centrifugation (Thermo Scientific Sorvall ST 16 Centrifuge) at room temperature using Ficoll solution. CD4+ T cells were collected using CD4 magnetic microbeads (130-045-101, Miltenyi Biotec) and then labeled with CFSE (C34554, Invitrogen) according to the manufacturer's protocol. MSCs from P3 or pretreated cells were then digested with trypsin and seeded in 24-well culture plates at a density of 2×104 cells/well (5:1) or 1×104 cells/well (10:1) with culture medium for 72h. CD4+ T cells from the coculture system were analyzed by flow cytometry to quantify the proportion of CFSE low cells.

The collected MSCs from different tissue origins were separately seeded into 75cm2 cell culture flask at the density of 1×106/cm2 with DMEM/F12 (11320033, GIBCO) medium plus 10% FBS, 2mM L-glutamine and 1% penicillin/streptomycin solution. Cells were cultivated at 37°C in a saturated humidified atmosphere containing 5% CO2 in CO2 Incubator (Thermo Fisher Scientific, Inc.). After 72 h, the non-adherent cells were removed by washing with PBS solution, after then the medium was changed every three days. The MSCs cultured to the density of 80% following treatment with 0.05% trypsin (27250018, GIBCO) and 0.02% EDTA (AM9912, Invitrogen) for 3 min at 37°C. The cells were washed by centrifugation at 300 g for 5 min via centrifugation (Thermo Scientific Sorvall ST 16 Centrifuge), then replanting to a new T75 cell culture flask at a lower density. MSCs were cultured to the third passage (P3) and digested for FC analysis.

Instrument

Flow cytometry data were acquired on FACS Canto II flow cytometer (BD Biosciences)

Software

The FlowJo software was used in FC data analysis.

Cell population abundance

The population abundance of PD-L1 positive MSCs were provided in Figure 4 , Supplementary Figure 5 and Figure 7, The population abundance of proliferating CD4+ T cells were provided in Supplementary Figure 4, Supplementary Figure 5, Supplementary Figure 7.

Gating strategy

The proportion of PD-L1-positive MSCs was calculated by comparing the same MSC sample labeled with PD-L1 FITC and the FITC isotype control, with detection at the same FITC channel voltage.

- [x] Tick this box to confirm that a figure exemplifying the gating strategy is provided in the Supplementary Information.
